# Manual exchange transfusion for severe imported falciparum malaria: a retrospective study

**DOI:** 10.1186/s12936-018-2174-z

**Published:** 2018-01-16

**Authors:** Jinfeng Lin, Xiaoying Huang, Gang Qin, Suyan Zhang, Weiwei Sun, Yadong Wang, Ke Ren, Junxian Xu, Xudong Han

**Affiliations:** 1 Department of Critical Care Medicine, Nantong Third People’s Hospital, Nantong University, 60 Mid-Youth Road, Nantong, 226006 China; 2 Center for Liver Diseases, Nantong Third People’s Hospital, Nantong University, 60 Mid-Youth Road, Nantong, 226006 China

**Keywords:** Severe imported falciparum malaria, Exchange transfusion, Liver function, Coagulation, Inflammation, Parasite clearance, Outcome

## Abstract

**Background:**

This study was designed to evaluate the efficacy of exchange transfusion in patients with severe imported falciparum malaria. Twelve patients who met the diagnostic criteria for severe malaria were treated with exchange transfusion 14 times according to a conventional anti-malarial treatment. This study evaluated the efficacy of exchange transfusion for severe imported falciparum malaria.

**Methods:**

Clinical data of severe imported falciparum malaria patients admitted to the intensive care unit (ICU) of Nantong Third People’s Hospital from January 2007 to December 2016 were investigated in this retrospective study. Patients were divided into the intervention group, which received exchange transfusion, and the control group. This study assessed parasite clearance and outcomes of the two groups, and levels of erythrocytes, haemoglobin, platelets, coagulation, liver function, lactate, C-reactive protein, and procalcitonin, before and after exchange transfusion in the intervention group.

**Results:**

There was no significant difference in the severity of admitted patients. Exchange transfusion was successfully applied 14 times in the intervention group. Differences in the levels of erythrocytes, haemoglobin and platelets did not reach statistical significance. Exchange transfusion improved coagulation, liver function, lactic acid, C-reactive protein, and procalcitonin. No differences were observed in parasite clearance, ICU and hospital length of stay, in-hospital mortality, and costs of hospitalization between the two groups.

**Conclusion:**

Exchange transfusion as adjunctive therapy for severe malaria was observed to be safe in this setting. Exchange transfusion can improve liver function and coagulation and reduce inflammation, but it failed to improve parasite clearance and the outcomes of severe imported falciparum malaria in this case series.

## Background

Many severe malaria patients who are admitted to an intensive care unit (ICU) have suffered from dysfunction in their liver, kidneys and other organs. The incidence of severe malaria was 10% in imported falciparum malaria. Advances in the care of severe imported falciparum malaria, including mechanical ventilation, blood purification and other treatments used to maintain organ function, have resulted in improved outcomes [1]. However, the mortality of imported falciparum malaria remains nearly 1% [2]. Asexual reproduction of *Plasmodium falciparum* occurs mainly in the erythrocytes. The high density of *Plasmodium* in severe malaria patients can lead to multiple organ dysfunction [3]. In this study, 12 patients who met the diagnostic criteria for severe malaria [4] were treated with exchange transfusion 14 times, as well as a conventional anti-malarial treatment. This study evaluated the efficacy of exchange transfusion for severe imported falciparum malaria.

## Methods

### Patient selection

The inclusion criteria were: (1) hospital admission from January 2007 to December 2016; (2) a diagnosis of falciparum malaria based on a blood smear performed at the hospital and the centres for disease control and prevention; and, (3) meeting the severe malaria diagnostic criteria [4], which were defined as cerebral malaria characterized by impaired consciousness (Glasgow Coma Scale score < 11) or convulsions, circulatory collapse, acute respiratory distress syndrome, jaundice, along with other organ dysfunction, haemoglobinuria, spontaneous bleeding, hypoglycaemia (plasma glucose < 2.2 mmol/L), severe anaemia (haemoglobin < 50 g/L or packed cell volume < 15%),metabolic acidosis (plasma bicarbonate < 15 mmol/L or pH < 7.35), hyperlactataemia (lactate > 5 mmol/L), acute kidney injury (plasma creatinine > 265 mmol/L), and *P. falciparum* parasitaemia > 10%.

### Patient assessments

Patients were divided into the intervention group, which received exchange transfusion, and the control group. This retrospective study was not random. Patients in the intervention group received exchange transfusion within 24 h of admission to the ICU. Admission clinical status was assessed using the Acute Physiology and Chronic Health Evaluation II score, Glasgow Coma Scale, levels of mean arterial pressure, plasma glucose, haemoglobin, plasma pH, lactate, creatinine, total bilirubin, pulmonary oedema, percutaneous oxygen saturation, respiratory rate and parasitaemia. This study compared the parasite clearance between the two groups and the laboratory values of the intervention group before and after exchange transfusion. Laboratory values were tested 8 h after exchange transfusion. Outcome measures, including length of ICU and hospital stay, in-hospital mortality and costs of hospitalization were compared at the same time.

### Treatment protocol

#### Basic treatment

Anti-malarial therapy with artesunate and clindamycin was initiated as soon as possible in all patients with malaria. Internal jugular vein and radial artery catheters were placed to obtain haemodynamic monitoring parameters. Continuous renal replacement therapy was used in patients with acute renal failure. Apheresis platelets were administered to patients with a platelet count < 20 × 10^9^/L. Heparin was used for systemic anticoagulation. Mechanical ventilation with low tidal volumes was used in patients with acute respiratory distress syndrome.

#### Exchange transfusion

Patients were eligible for exchange transfusion when they met one of the following criteria: *Plasmodium* trophozoites found in peripheral blood; pH < 7.25; or, diagnosis of disseminated intravascular coagulation. Similar to prior reports [5], exchange transfusion was performed using a manual isovolumetric approach. The exchange transfusion protocol was executed as follows: a catheter was inserted in a peripheral vein, which was used to transfuse the packed red cell suspensions and plasma or 20% albumin (the ‘in-flow’ tract), and another catheter was inserted into the flexible artery, which was used to collect blood into a donor set (the ‘out-flow’ tract). 500 mL of blood was collected from the out-flow tract. Saline was provided via the in-flow catheter at the same time. The rate of saline transfusion was controlled to approximate the blood extracted from the out-flow tract. Two hours later, one unit of packed red cell suspension (approximate volume 200–250 mL with a haematocrit of 0.5–0.65) and one unit of fresh-frozen plasma (volume approximately 280 mL) or 150 mL 20% albumin was used as the replacement solution by the in-flow tract. The volume of the exchange procedure was 500 mL of whole blood every time. The frequency of the exchange procedure depended on parasite count and the level of haemoglobin. The aim of the exchange procedure was that the parasite count dropped below 1 × 10^5^ parasite/μL. The exchange procedure ceased when the level of haemoglobin had dropped below 70 g/L. The volume of the exchange was 500–1000 mL in most patients. Mean arterial pressure, pulse rate and oxygen saturation were monitored frequently during the exchange transfusion procedure.

### Statistical analysis

Data were analysed using SPSS software for Windows, version 17.0 (SPSS, Chicago, IL, USA). Continuous data are presented as the mean ± standard deviation. An independent sample *t* test was performed to compare continuous variables. Differences before and after exchange transfusion were assessed using a paired sample *t* test. Categorical variables were compared using the Fisher’s exact test. A *P* value of < 0.05 was considered to be the level of significance.

## Results

### Clinical status of patients at admission

A total of 27 patients (12 control group and 15 intervention group) were eligible according to the inclusion criteria. All the patients were male and had working experience in Africa. No differences were observed in age, APACHE II score, Glasgow Coma Scale and levels of mean arterial pressure, plasma glucose, haemoglobin, plasma pH, lactate, creatinine total bilirubin, pulmonary oedema, percutaneous oxygen saturation, respiratory rate, and parasitaemia between the two groups. There was no significant difference in the severity of admitted patients. The details are shown in Table 1.

**Table 1:** Admission clinical status

	Age (year)	APACHE II score	Glasgow Coma Scale	Mean arterial pressure (mmHg)	Plasma glucose (mmol/L)	Haemoglobin (g/L)	Plasma pH
Exchange transfusion	47.8 ± 4.4	15.00 ± 5.98	11.33 ± 3.60	68.67 ± 21.23	6.26 ± 2.13	80.5 ± 28.5	7.37 ± 0.77
Control	46.0 ± 5.8	12.93 ± 4.93	10.73 ± 3.26	75.93 ± 18.59	5.73 ± 2.10	86.6 ± 23.1	7.38 ± 0.88
*P*	0.397	0.334	0.654	0.352	0.523	0.544	0.654

	Lactate (mmol/L)	Creatinine (µmol/L)	Total bilirubin (µmol/L)	Pulmonary oedema	Percutaneous oxygen saturation (%)	Respiratory rate (breaths/min)	Parasitaemia (10^5^ parasite/μL)
Exchange transfusion	5.5 ± 2.7	203 ± 98	114.02 ± 75.73	3 (25.0%)	97.92 ± 3.50	16.17 ± 3.17	7.63 ± 5.28
Control	4.3 ± 3.4	184 ± 115	103.74 ± 54.11	2 (13.3%)	96.93 ± 3.26	15.40 ± 3.40	5.71 ± 2.52
*P*	0.313	0.648	0.684	0.628	0.458	0.555	0.224

### Parasite clearance between the two groups

The parasite clearance is shown in Table 2. No differences in parasite density at admission were observed between the two groups. After the initiation of anti-malarial therapy, it is observed that a rapid decrease in parasite counts. However, exchange transfusion did not contribute significantly to parasite clearance.

**Table 2:** Parasitaemia variables (10^5^ parasite/μL)

	0	12 h	24 h	36 h
Exchange transfusion	7.42 ± 5.18	1.58 ± 1.88	0.50 ± 0.90	0.08 ± 0.29
Control	5.87 ± 2.64	1.20 ± 1.57	0.20 ± 0.56	0
*P*	0.322	0.568	0.300	0.272

### Laboratory values of the intervention group before and after exchange transfusion

Exchange transfusion was safely performed 14 times in the intervention group. No significant changes in white blood cell count, haemoglobin and platelet count were detected. There was no significant difference in the levels of prothrombin time, activated partial thromboplastin time, fibrinogen and antithrombin III after exchange transfusion. Exchange transfusion reduced the levels of fibrin degradation products and D-dimer as well as improving liver function (reduced levels of aspartate aminotransferase, total bilirubin and lactate dehydrogenase but not alanine aminotransferase). At the same time, exchange transfusion reduced levels of lactate, C-reactive protein and procalcitonin. The details are shown in Table 3.

**Table 3:** Laboratory values of intervention group before and after exchange transfusion

	WBC (10^9^/L)	Hb (g/L)	PLT (10^9^/L)	PT (S)	APTT (S)	FIB (g/L)	AT III (%)	FDP (mg/L)
Before exchange transfusion	8.89 ± 3.48	104.14 ± 18.35	33.83 ± 14.15	13.69 ± 1.91	56.09 ± 10.83	2.70 ± 0.90	88.71 ± 14.13	56.12 ± 42.21
After exchange transfusion	8.32 ± 3.21	98.43 ± 16.35	38.21 ± 11.09	13.22 ± 1.41	46.38 ± 11.00	2.68 ± 0.66	86.06 ± 16.52	23.67 ± 30.62
*P*	0.443	0.071	0.175	0.447	0.345	0.838	0.364	0.001

	DD (mg/L)	ALT (u/L)	AST (u/L)	TBI (µmol/L)	LDH (u/L)	LAC (µmol/L)	CRP (mg/L)	PCT (ng/ml)
Before exchange transfusion	34.15 ± 24.52	67.14 ± 35.88	104.93 ± 58.32	100.43 ± 74.25	1187.4 ± 558.9	3.04 ± 1.10	155.21 ± 69.10	53.83 ± 29.41
After exchange transfusion	14.34 ± 15.17	55.57 ± 35.76	76.07 ± 27.74	62.96 ± 48.54	713.0 ± 314.5	2.10 ± 0.65	88.86 ± 36.83	24.71 ± 15.82
*P*	0.001	0.097	0.025	0.002	0.002	0.001	0.001	0.00

*WBC* white blood cell count, *Hb* haemoglobin, *PLT* platelet, *PT* prothrombin time, *APTT* activated partial thromboplastin time, *FIB* fibrinogen, *AT III* antithrombin III, *FDP* fibrin degradation products, *DD D-dimer*, *ALT*, alanine aminotransferase, *AST* aspartate aminotransferase, *TBI* total bilirubin, *LDH* lactate dehydrogenase, *LAC* lactate, *CRP* C-reactive protein, *PCT* procalcitonin

### Outcome

The length of ICU stay was 8.8 ± 5.0 days for the control group *versus* 7.3 ± 4.7 days for the intervention group. The length of hospital stay was 14.7 ± 6.7 days for the control group *versus* 16.7 ± 7.7 days for the intervention group. No case fatalities were observed in the intervention group, whereas two deaths (a mortality rate of 13.3%) were noted in the control group (p values more than 0.05; Fisher’s exact test). The costs of hospitalization were 10,6135 ± 54,092 yuan for the control group *versus* 86,165 ± 37,137 yuan for the intervention group. However, there were no statistically significant differences between the two groups for the lengths of ICU and hospital stays, in-hospital mortality or costs of hospitalization (all *p* values more than 0.05). Exchange transfusion failed to improve outcomes. This result may be biased due to the small sample size.

## Discussion

Severe malaria is a sub-set of malaria with high mortality rates. Most severe malaria is caused by falciparum malaria. Falciparum malaria is extremely common in Africa. Severe malaria mostly occurs in children and women in endemic areas. In recent years, there are many cases of imported falciparum malaria in China. In the early stages of severe malaria, there are no specific clinical symptoms, which results in delayed diagnosis and incorrect treatments.

All the patients in this study had working experience in Africa. They were male and previously healthy. Most patients were haemodynamically stable when admitted to the ICU. One early symptom was an unexplained fever in many patients. Because of delayed diagnosis, many patients had developed hyperbilirubinemia, kidney injury, active coagulation, and haemolysis when malaria was diagnosed.

*Plasmodium* can cause erythrocyte destruction and adherence to endothelial cells, which leads to microcirculatory dysfunction [6]. Removing infected red blood cells and plasma (including parasitic antigens and toxic products) is theoretically effective for treating severe falciparum malaria.

Exchange transfusion was first introduced in 1974 [7] as an adjunctive therapy for the treatment of severe malaria. Although exchange transfusion was successfully used in numerous cases [8, 9], its use remains controversial. Exchange transfusion was associated with risks of fluid overload, transfusion reactions and potential transmission of blood-borne infections [10]. No sufficiently powered, randomized, controlled study has been reported. A meta-analysis found that exchange transfusion failed to improve the survival rate [11]. Another study found that the parasite clearance times were significantly shorter for patients treated with exchange transfusion compared to patients treated with only parenteral quinine [5]. Kreeftmeijer-Vegter found that exchange transfusion could not significantly contribute to parasite clearance in artesunate-treated individuals but could improve parasite clearance under quinine treatment [10].

This research focused on parasite clearance. In severe malaria, infected red blood cells have decreased deformability and increased adhesiveness, which caused sequestration of these cells in the microvasculature, local hypoxia, ischaemia, and local release of pro-inflammatory cytokines [12]. Exchange transfusion removes circulating, infected red blood cells and toxic by-products, which theoretically should improve microcirculation. The UK has considered the application of automated red blood cell exchange in their malaria treatment guidelines and recommends its use for patients with high parasitaemia (> 10% infected red blood cells) and organ failure [13].

In this study, exchange transfusion was safely applied 14 times. Research involving the role of exchange transfusion in improving liver function and coagulation as well a reducing inflammation in severe imported falciparum malaria patients remains rare. According to this findings, exchange transfusion can improve liver function and coagulation as well as reduce inflammation, but it failed to improve parasite clearance, which is consistent with findings from previous studies [10]. This retrospective study was limited due to the small number of included patients. A randomized controlled trial is needed to unequivocally establish whether exchange transfusion is beneficial.

## Conclusion

This study suggests that using exchange transfusion as an adjunctive therapy for severe malaria was safe in this setting. Exchange transfusion can improve liver function and coagulation as well as reduce inflammation, but it failed to improve parasite clearance and outcomes of severe imported falciparum malaria in this case series.
